# 624 How We Do It: Schedule Operating Room Nursing Staff to Maintain Burn Surgery Competency

**DOI:** 10.1093/jbcr/iraf019.253

**Published:** 2025-04-01

**Authors:** Sheiryl Gica, Shannon Caesar-Peterson, Abraham Houng

**Affiliations:** New York Presbyterian Hospital - Weill Cornell; New York Presbyterian Hospital - Weill Cornell; New York Presbyterian Hospital - Weill Cornell

## Abstract

**Introduction:**

Burn surgery is a unique and distinct surgical specialty as compared with other surgeries in the operating room. Having dedicated nursing staff in the operating room who understand the intricacies of burn surgeries can be invaluable. Dedicated nursing staff can help the surgeon expedite the procedure. However, as a level 1 trauma center and a verified burn center, all operating room nursing staff, regardless of expertise, should be competent in burn surgery in the event of a mass-casualty incident. Allowing all operating room nursing staff the opportunity to participate in burn surgeries might negate the benefit of having dedicated nursing staff, but it will ensure all nursing staff have a baseline of knowledge and competency in burn surgery.

**Methods:**

We propose using Benner’s Novice to Expert Nursing Theory as the framework and systematic method of having nursing staff from various services rotated in burn surgeries, either as a scrub nurse or in the circulator role. Dedicated burn operating room nursing staff ensures other staff member who has never been assigned in burns are given an orientation. The dedicated nursing staff serves as mentors in the operating room. Once the nursing staff have gained some mastery and competency in burn surgeries, they may function independently in the burn operating room.

**Results:**

The following is the number of operating room nursing staff that participated in the training: 53% (37). 100% (10) of newly hired operating room nursing staff regardless of specialty are fully trained in burns during orientation period. They were trained by dedicated burn operating staff which comprised of 3 nurses and 2 surgical technicians. Approximately 80% of burn surgeries in the last year were staffed by dedicated burn staff, and 20 % of burn surgeries were staffed by other service nurses. 90% of all operating room nursing staff are given in-service of the latest innovation and updates in burn surgery.

**Conclusions:**

Having all operating room nursing staff be competent in burn surgery is a must in an event of a mass casualty. We need to apply Benner’s Novice to Expert Nursing Theory to help the nursing staff acquire and maintain their competencies; for example, having the competent nursing staff rotate to burn every 3 months to refresh their burn surgery knowledge. There are other questions that arise from this study: are we sacrificing care by not having dedicated burn nursing staff cover all burn cases? Is there a difference in patient outcome? These are ideas that we will study in the future.

**Applicability of Research to Practice:**

Applicable to clinical practice.

**Funding for the Study:**

N/A

